# Cancer-Associated Fibroblasts in Prostate Cancer: Unraveling Mechanisms and Therapeutic Implications

**DOI:** 10.32604/or.2025.073265

**Published:** 2026-01-19

**Authors:** Yang Wu, Dong Xu, Run Shi, Mingwei Zhan, Shaohui Xu, Xin Wang, Jianpeng Zhang, Zhaokai Zhou, Weizhuo Wang, Yongjie Wang, Minglun Li, Zihao Xu, Kaifeng Su

**Affiliations:** 1 The First Clinical School, The First Affiliated Hospital of Nanjing Medical University, Nanjing, 210000, China; 2 Department of Urology, Hangzhou TCM Hospital of Zhejiang Chinese Medical University (Hangzhou Hospital of Traditional Chinese Medicine), Hangzhou, 310007, China; 3 Institute of Functional Nano & Soft Materials (FUNSOM), Soochow University, Suzhou, 215123, China; 4 Department of Oncology, Jiangsu Cancer Hospital, Nanjing, 210009, China; 5 Department of Urology, The First Affiliated Hospital of Guangzhou Medical University, Guangzhou, 510030, China; 6 Department of Urology, The Second Xiangya Hospital of Central South University, Changsha, 410011, China; 7 Center for Reproductive Medicine, The Second Affiliated Hospital of Soochow University, Suzhou, 215000, China; 8 Proteomics and Cancer Cell Signaling Group, German Cancer Research Center (DKFZ), Heidelberg, 69120, Germany; 9 Department of Radiation Oncology, Lueneburg Municipal Hospital, Lueneburg, 21339, Germany; 10 Department of Urology, The First Affiliated Hospital of Nanjing Medical University, Nanjing, 210029, China; 11 The First Affiliated Hospital of Shandong First Medical University & Shandong Provincial Qianfoshan Hospital, Jinan, 250013, China

**Keywords:** Prostate cancer, cancer-associated fibroblasts, tumor microenvironment, therapy resistance

## Abstract

Prostate cancer (PCa) remains a major cause of cancer-related mortality in men, largely due to therapy resistance and metastatic progression. Increasing evidence highlights the tumor microenvironment (TME), particularly cancer-associated fibroblasts (CAFs), as a critical determinant of disease behavior. CAFs constitute a heterogeneous population originating from fibroblasts, mesenchymal stem cells, endothelial cells, epithelial cells undergoing epithelial–mesenchymal transition (EMT), and adipose tissue. Through dynamic crosstalk with tumor, immune, endothelial, and adipocyte compartments, CAFs orchestrate oncogenic processes including tumor proliferation, invasion, immune evasion, extracellular matrix remodeling, angiogenesis, and metabolic reprogramming. This review comprehensively summarizes the cellular origins, phenotypic and functional heterogeneity, and spatial distribution of CAFs within the prostate TME. We further elucidate the molecular mechanisms by which CAFs regulate PCa progression and therapeutic resistance, and critically evaluate emerging strategies to therapeutically target CAF-mediated signaling, metabolic, and immune pathways. By integrating recent advances from single-cell and spatial transcriptomics (ST), our objective is to provide a holistic framework for understanding CAF biology and to highlight potential avenues for stromal reprogramming as an adjunct to current PCa therapies.

## 1 Introduction

According to 2022 estimates by the International Agency for Research on Cancer, prostate cancer (PCa) accounted for 1,466,680 new cases (7.3% of all malignancies) and 396,792 deaths (4.1% of cancer-related mortality) [1]. Established risk factors include advanced age, African ancestry, and a familial predisposition to PCa [2]. While localized PCa exhibits a high long-term survival, metastatic disease remains incurable, mainly due to multimodal therapies [3]. PCa progression is primarily regulated by androgen signaling pathways, which are mediated through the activation of the androgen receptor (AR) [4]. Clinical management stratifies patients by risk profile, encompassing options from active surveillance to radical prostatectomy, radiotherapy, and androgen deprivation therapy (ADT) [2]. Tumors frequently develop therapeutic resistance, progressing to castration-resistant prostate cancer (CRPC) characterized by prostate-specific antigen (PSA) elevation despite castrate serum testosterone levels [5,6]. Critical knowledge gaps in PCa molecular pathogenesis and the limited efficacy of current therapies for advanced disease underscore the urgent need for mechanistic investigations [7]. Emerging evidence highlights the tumor microenvironment (TME) as a key mediator of therapeutic resistance, metastatic dissemination, and disease progression in PCa.

TME comprises non-epithelial cellular components (e.g., fibroblasts, immune cells, endothelial cells) and extracellular matrix (ECM) constituents such as collagen, laminin, fibronectin, and hyaluronate [8]. Distinct immune cell infiltrates exhibit pro-tumorigenic properties and demonstrate a significant association with decreased PSA recurrence-free survival [9]. Chronic immune infiltration induces stromal remodeling through the recruitment and epigenetic reprogramming of cancer-associated fibroblasts (CAFs) and a newly characterized population of metastasis-initiating cells, which collectively enhance metastatic potential and therapeutic resistance [10]. Bidirectional crosstalk between neoplastic cells and TME elements, mediated by membrane receptors, growth factors, and matrix remodeling enzymes, orchestrates therapeutic resistance and metastatic dissemination [11]. Among all the TME elements, CAFs have emerged as pivotal regulators of tumor-stroma interactions. Deciphering CAF-mediated molecular pathways in prostate carcinogenesis offers promising therapeutic opportunities for targeting therapy-resistant PCa.

The primary objective of this review is to systematically elucidate the multifaceted roles of CAFs in PCa, with a particular focus on their cellular origins, phenotypic and functional heterogeneity, and the molecular mechanisms underlying their interactions within the TME. By integrating recent insights from single-cell and spatial transcriptomic technologies, this article aims to construct a comprehensive framework for understanding CAF-mediated regulation of tumor progression, metastasis, and therapeutic resistance. Furthermore, it critically evaluates emerging therapeutic strategies targeting CAF-derived signaling, metabolic, and immunomodulatory networks.

## 2 CAFs in Prostate Cancer

CAFs in prostate cancer originate from multiple cellular sources and exhibit diverse functions within the TME. This diversity in origin contributes to functional variability, highlighting the importance of detailed examination of their cellular sources.

### 2.1 Cellular Origins of CAFs in PCa

CAFs are defined as functionally heterogeneous stromal cells within the TME that exhibit distinct phenotypic and secretory profiles [12,13]. Multiple studies have identified multipotent precursors, including resident fibroblasts, mesenchymal stem cells (MSCs), endothelial cells, epithelial cells undergoing epithelial-to-mesenchymal transition (EMT), and adipose tissue as CAF progenitors.

#### 2.1.1 Resident Fibroblasts as the Primary Source

Resident fibroblasts are recognized as the principal source of CAFs [14,15]. Similar to wound healing mechanisms, transforming growth factor β (TGF-β)-mediated myofibroblast activation occurs during PCa progression [16,17]. They can evade apoptosis under the influence of growth factors and cytokines and then evolve into CAFs [16,17]. Also, senescent fibroblasts secrete paracrine factors that enhance prostate epithelial proliferation [18]. Hypoxia-induced fibroblasts demonstrate upregulation of α-smooth muscle actin (α-SMA) and secrete more collagen [19]. The Prostate Cancer Prevention Trial demonstrated that a reduction in telomere length of normal stromal cells is associated with an increased PCa risk [20]. Cumulative evidence suggests senescent fibroblasts represent plausible CAF progenitors [21].

#### 2.1.2 Mesenchymal Stem Cells (MSCs)

*In vivo* PCa models demonstrate that the C-X-C motif chemokine ligand 16 (CXCL16)—C-X-C motif chemokine receptor 6 (CXCR6) axis-mediated chemotaxis recruits MSCs from other organs, which subsequently undergo CAF differentiation within tumor stroma [21]. TGF-β1 stimulation induces similar MSC differentiation into CAFs, characterized by the expression of markers such as α-SMA, fibroblast activation protein (FAP), platelet-derived growth factor receptor β (PDGFRβ), and vascular endothelial growth factor receptor 2 (VEGFR2) [22]. In addition to MSCs from other organs, local mesenchymal cells have also been proven to have the potential to differentiate into CAFs. In stroma and epithelial cells, paracrine factors, including fibroblast growth factor (FGF), PDGFRα/β, TGF-β, epidermal growth factor (EGF), and insulin-like growth factor (IGF) family receptors, are upregulated, which are closely associated with mesenchymal cells [23]. Single-cell RNA sequencing (scRNA-seq) analysis of 12,346 stromal cells identifies PDGFRα^+^ CAF clusters spanning the progression stages of PCa, confirming that CAFs can originate from MSCs [24].

#### 2.1.3 Endothelial Cells

Endothelial cells exhibit phenotypic plasticity through endothelial-to-mesenchymal transition (EndMT), which represents a significant contributing source of CAFs in multiple malignancies [25]. TGF-β1 stimulation induces EndMT in pulmonary and cardiac endothelial cells [26,27]. Although there is no direct evidence to prove that CAFs can originate from MSCs, emerging results from recent studies are strengthening the possibility of this hypothesis. The scRNA-seq technology identifies CAF subsets that co-express endothelial markers, including cluster of differentiation 31 (CD31) and nidogen-2 (NID2), in breast tumors. Most of them are located near vasculature, indicating a possible endothelial origin [28]. Soon, additional work will be necessary to investigate whether prostate endothelial cells can differentiate into CAFs.

#### 2.1.4 Epithelial-to-Mesenchymal Transition (EMT)

EMT is a differentiation process that occurs from epithelial cells to motile mesenchymal cells, taking place during development, wound repair, and in the behavior of stem cells [29]. Its activation correlates with increased invasion capacity and resistance to apoptosis, contributing to the progression of carcinogenesis and metastasis [30,31]. Specifically, stemness and drug resistance of PCa are promoted through EMT, making it more progressive and resistant to therapy [32]. CAFs demonstrate EMT-associated signatures with co-expression of αSMA, fibroblast-specific protein 1 (FSP1), vimentin, and desmin [33]. Therefore, some CAFs may differentiate from mesenchymal cells, originating from epithelial cells.

#### 2.1.5 Adipose Tissue

*In vitro* experiments show that the differentiation of human adipose tissue-derived stem cells can be observed in breast cancer [34]. The proximity of breast cancer cells and adipocytes, driven by local invasion, induces adipocytes to undergo phenotypic changes, resulting in the formation of fibroblast-like cells [35]. A study conducted a Cox regression analysis and concluded that periprostatic adipose tissue invasion is a significant independent predictor of biochemical recurrence (HR 1.53, *p* = 0.018) [36]. These evidences propose a possible hypothesis that periprostatic adipose tissues may interact with PCa cells and transform into CAFs once extracapsular invasion occurs.

In general, CAFs have five principal origins: fibroblasts, MSCs, endothelial cells, EMT, and adipose tissues. While resident fibroblasts constitute the predominant CAF source, additional researches are needed to verify other currently uncertain origins. A better understanding of the CAF’s origin may offer new perspectives on the development of therapeutic strategies.

### 2.2 Phenotypic and Functional Heterogeneity of CAFs

The cellular heterogeneity of CAFs, stemming from their diverse cellular origins, makes it difficult to characterize them with a single marker. Different subtypes may express distinct combinations of markers and play distinct roles in the TME. So far, there is no general agreement on the classification of CAF subtypes. A functional classification system stratifies CAFs into five molecularly distinct subtypes (F1-F5) [16]. The tumor-restraining (F1 subtype) and the tumour-promoting (F2 subtype) CAFs are different and possess the ability to interconvert under specific conditions. Secretory (F3 subtype) CAFs can influence tumour immunity, angiogenesis, and cancer cell proliferation or metastasis through growth factor secretome activity. ECM remodelling (F4 subtype) can alter the ECM composition in the TME, elevating ECM stiffness or facilitating invasion. Besides, other related functions of CAFs are attributed to the F5 subtype. Some researchers summarize previous studies and define the subtype of prostate CAFs based on their protein and mRNA expression markers [37]. The CD90^+^subtype expressing CD90, Asporin (ASPN), vascular endothelial growth factor (VEGF), FGF2, patched 1 (PTCH1), TGF-β, and Interleukin 6 (IL6) are identified to drive tumor initiation. Both FGF2^+^ and HGF^+^ subtypes can influence ECM remodeling, which can be attributed to the F4 subtype we mentioned above. Another detailed subtype, along with its markers, functions, and cell of origin, can be acquired through the summary table. A scRNA-seq study has classified CAFs into six distinct subtypes (CAF-0 to CAF-5) [38]. Among these subtypes, C-C motif chemokine ligand 2 (CCL2)-expressing CAF-0 recruits tumor-associated macrophages (TAMs), whereas CXCL12-producing CAF-1 attracts immune cells, including mast cells, innate lymphoid cells, and eosinophils. These subtypes collectively foster an immunosuppressive TME in PCa. An integrated analysis of RNA-seq and ChIP-seq data further demonstrates that the CCL2/CXCL8 cytokines mediate CAF-driven migration and invasion in PCa cells [39]. Advanced transcriptomic profiling enables more precise subtyping and functional characterization of CAFs. Current function-based classification methods lack the precision required for molecular mechanistic studies. Marker-dependent classification utilizes non-specific protein/mRNA signatures that overlap with those of myofibroblasts and macrophages in specific disease contexts [40,41]. However, single-cell transcriptomics offers a transformative framework for refining CAF classification systems.

### 2.3 Recent Advances in the Spatial Transcriptomics

In recent years, spatial transcriptomics (ST) has begun to shed light on the spatial heterogeneity, neighborhood relationships, and functional specialization of CAFs within the prostate TME.

Using an integrative single-cell spatial multi-omics framework across multiple tumor types and platforms, Zhang et al. identified four spatially conserved CAF subtypes with distinct molecular programs and ecological niches [42]. Specifically, s1-CAFs were characterized by contractile and extracellular matrix–remodeling signatures and localized adjacent to tumor cells, while s2-CAFs exhibited high IL6 expression and inflammatory features within interstitial regions. s3-CAFs preferentially neighbored vascular and myeloid compartments and expressed stress- and complement-associated genes, correlating with immune exhaustion and poor immunotherapy response. In contrast, s4-CAFs were enriched near tertiary lymphoid structures and displayed antigen-presenting and chemokine signatures that potentially facilitate T/B cell recruitment and activation.

Collectively, this study provides a spatially resolved taxonomy of CAFs and highlights novel microenvironmental targets for therapeutic intervention.

## 3 CAF-Mediated Cellular Interactions within the Prostate Cancer TME

CAFs have been shown to play a crucial role in the TME. Multiple interactions between CAFs and other cells in TME, including tumor cells, immune cells, endothelial cells, and adipocytes, orchestrate key oncogenic processes in PCa, encompassing disease progression, metastatic dissemination, and therapy resistance.

### 3.1 CAF-Tumor Cell Crosstalk and Its Oncogenic Consequences

**Figure 1:**
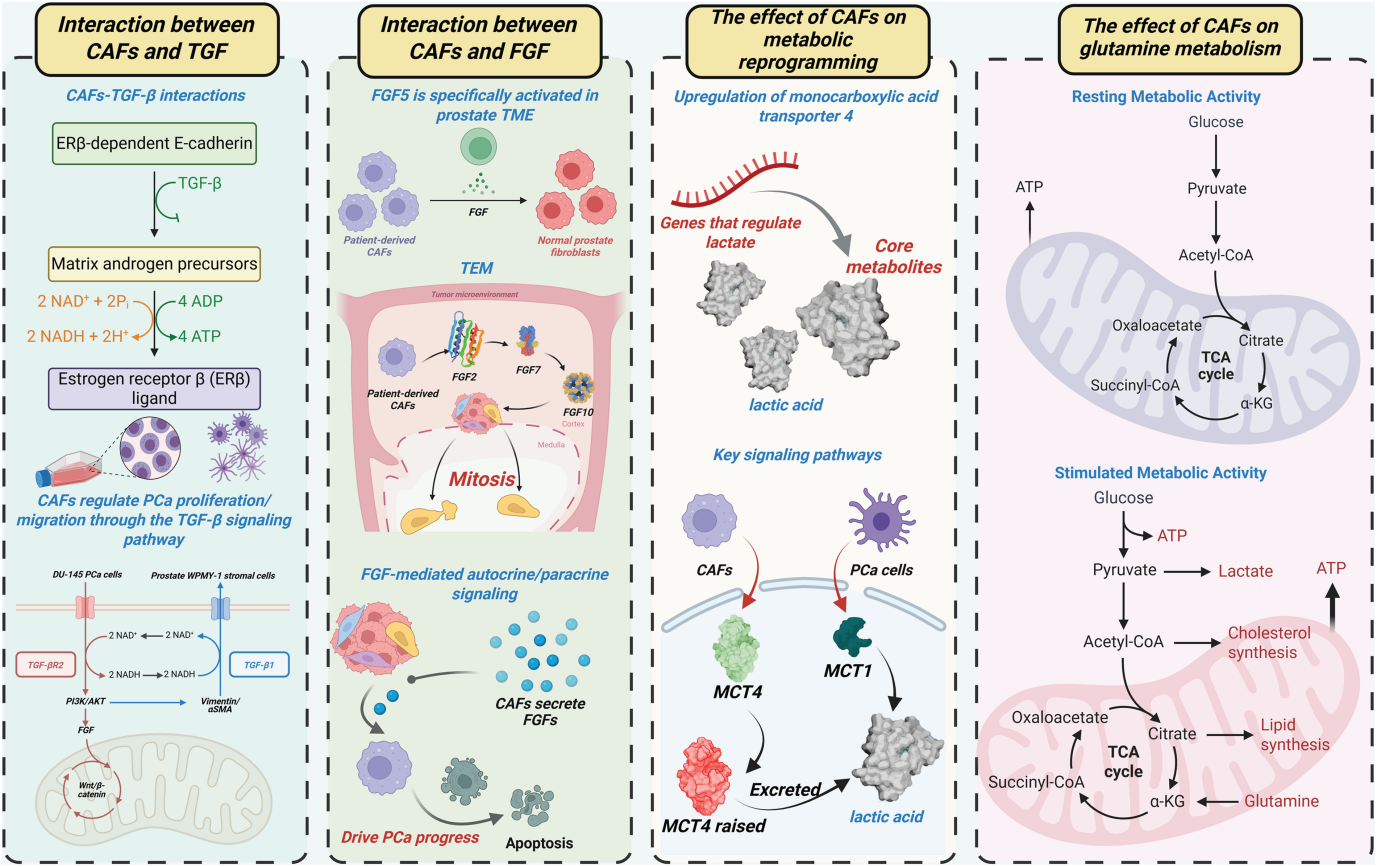
CAFs orchestrate PCa progression via multicellular crosstalk. This figure summarizes the key mechanisms involving CAFs in the prostate tumor microenvironment (TME), including their interactions with TGF-β and FGF signaling pathways, and their role in regulating metabolic activity. The figure illustrates how CAFs influence PCa progression through autocrine and paracrine signaling, and how they modulate key metabolic pathways such as lactate and glutamine metabolism in both resting and stimulated metabolic states. CAFs: Cancer-associated fibroblasts; PCa: Prostate cancer; TME: Tumor microenvironment; TGF-β: Transforming growth factor beta; FGF: Fibroblast growth factor; ERβ: Estrogen receptor beta; MCT: Monocarboxylate transporter; ATP: Adenosine triphosphate; NAD+: Nicotinamide adenine dinucleotide; NADH: Reduced nicotinamide adenine dinucleotide; CoA: Coenzyme A; α-KG: Alpha-ketoglutarate; TEM: Tumor microenvironment; PI3K: Phosphatidylinositol 3-kinase; AKT: Protein kinase B; TCA: Tricarboxylic acid cycle

CAFs secrete a diverse array of bioactive factors that promote PCa progression. They can enhance PCa cell proliferation and invasiveness by secreting growth factors and facilitating metabolic reprogramming (Fig. 1). Epigenetic reprogramming further contributes to these oncogenic processes. Besides, CAFs confer therapy resistance, particularly to ADT and chemotherapy, through multimodal mechanisms.

#### 3.1.1 Growth Factor Signaling: TGF-β and FGF Pathways

TGF-*β* and FGF represent the most extensively studied growth factors in this context. Co-culture of human DU-145 PCa cells with prostate WPMY-1 stromal cells revealed metabolic conversion of stromal androgenic precursors to estrogen receptor β (ERβ) ligands [43]. This process involves TGF-β-mediated suppression of ERβ-dependent E-cadherin transcription, enhancing cancer cell motility. CAFs regulate PCa proliferation/migration through TGF-β signaling, as demonstrated in prior mechanistic studies [44]. Across malignancies (e.g., gastric, lung, breast cancers), CAFs employ TGF-β-enriched paracrine signaling to drive tumor growth and invasion [45–47]. Research focusing on PCa has extensively characterized the crosstalk between CAFs and TGF-β, as well as its synergistic regulation of tumor phenotypes. TGF-β1 upregulation induces vimentin/αSMA expression and drives benign prostatic hyperplasia (BPH) cell transformation, generating aggressive tumor phenotypes *in vivo* [48]. These TGF-β-mediated effects mirror the characteristic functions of CAFs. We propose a feedforward mechanism where TGF-β activates CAFs, which subsequently secrete additional TGF-β to amplify prostate carcinogenesis. CAFs utilize autocrine TGF-β signaling for self-maintenance and paracrine signaling for microenvironmental crosstalk [49,50]. However, clinical analyses reveal that TGF-β receptor 2 (TGF-βR2) is lost in 69% of malignant vs. 15% of benign prostate stroma, indicating the presence of TGF-β-independent CAF subsets [49]. Furthermore, TGF-βR2-deficient CAFs accelerate prostate cancer progression [49]. Therefore, TGF-βR2 loss in CAFs represents a distinct oncogenic mechanism warranting further investigation. TGF-β acts as a signaling hub that coordinates CAF activity through integration of PI3K/AKT, FGF, and Wnt/β-catenin pathways, collectively promoting tumor progression and survival [51–55].

The FGF family comprises 22 structurally related proteins regulating fundamental biological processes, including tissue growth, morphogenesis, development, and repair [56]. Among 18 canonical secreted FGFs, FGF5 demonstrates specific activation within the prostate TME [57]. Comparative analysis of patient-derived CAFs vs. matched normal prostate fibroblasts revealed elevated FGF5 expression in CAFs [58]. CAFs additionally express FGF2, FGF7, and FGF10, as validated across multiple studies [59,60]. These FGF ligands induce potent mitogenic activation in epithelial compartments [61]. Mirroring TGF-β functionality, CAFs employ FGF-mediated autocrine/paracrine signaling to coordinate TME cellular crosstalk. To be more specific, CAF-derived FGFs activate oncogenic pathways (RAS/MAPK, PI3K/AKT/mTOR, JAK/STAT) that drive prostate cancer progression [62,63].

#### 3.1.2 Metabolic Reprogramming

Beyond growth factor signaling, metabolic reprogramming has a critical influence on PCa progression. Lactate functions as a key metabolic mediator facilitating CAF-PCa cellular crosstalk [64]. Activated CAFs in the TME upregulate monocarboxylate transporter 4 (MCT4) to enhance lactate efflux. Concurrently, PCa cells increase MCT1-mediated lactate uptake, utilizing this metabolite to fuel proliferation under glucose-deprived conditions. Therefore, elevated MCT4 expression in the PCa TME correlates with adverse clinical outcomes [65,66]. Glutamine metabolism represents another crucial axis of PCa metabolic reprogramming. Epigenetic silencing of RASAL3 (a Ras GTPase-activating protein) in CAFs activates oncogenic Ras signaling, promoting macropinocytosis-dependent glutamine production [67]. This glutamine is transported via SLC1A5 into PCa cells to fuel tumor proliferation. CAF-derived lactate upregulates lipid metabolism genes in PCa cells, driving lipid droplet accumulation and supplying acetyl moieties for histone acetylation [68]. These findings establish a metabolite-epigenetic axis in cancer progression.

#### 3.1.3 Epigenetic Regulation and Therapeutic Potential

It is plausible—and in part supported by emerging literature—that the epigenetic status of CAFs in PCa shapes their functional phenotypes, and that therapeutically modulating CAF epigenetics may offer a route to reprogram the tumor stroma in PCa. First, although direct studies of CAF epigenetics in PCa remain limited, pan-cancer integrative analyses have revealed that CAFs across tumor types (including prostate) harbor conserved aberrant DNA methylation landscapes compared to normal fibroblasts. For instance, an integrative analysis including PCa datasets showed recurrent differential CpG methylation in CAFs relative to normal fibroblasts, and that such methylation changes correlate with gene expression alterations [69]. This suggests that analogous epigenetic reprogramming may occur in prostate CAFs, potentially underpinning pro-tumorigenic gene expression programs. Histone acetylation readers (e.g., bromodomain and extraterminal [BET] proteins) emerge as promising therapeutic targets, with BET inhibitors demonstrating clinical efficacy across malignancies [70]. BET protein inhibition attenuates the metastatic potential of lactate-reprogrammed PCa cells. Second, there is mechanistic plausibility for epigenetic control of CAF functions (e.g., secretion of growth factors, ECM remodeling, immunomodulation). It has been reported that DNA methylation changes and histone modifications during CAF maturation can lock in transcriptional states that maintain the activated fibroblast phenotype [71].

This CAF-mediated epigenetic regulation represents an emerging therapeutic frontier requiring further exploration.

#### 3.1.4 CAF-Induced Therapeutic Resistance Mechanisms

AR signaling inhibitor-based ADT constitutes the first-line treatment for PCa with postoperative progression, radiotherapy failure, or an initial presence of metastases [4]. However, therapeutic resistance invariably develops during progression to CRPC. Compelling evidence suggests that CAFs compromise the efficacy of AR-targeted therapy through multiple mechanisms. Three-dimensional spheroid co-culture models reveal CAF-mediated resistance to AR antagonists (enzalutamide, bicalutamide) in PCa cell lines (DuCaP, LNCaP, LAPC4) [72]. Mechanistically, CAFs induce PI3K/AKT pathway activation in tumor cells, bypassing androgen dependency. CAF-secreted HMGCS2 (3-hydroxy-3-methylglutaryl-CoA synthase 2) and AKR1C3 (aldo-keto reductase 1C3) enhance cholesterol metabolism and steroidogenesis, establishing AR-independent survival pathways [73]. ADT attenuates CAF-derived exosomal miR-146a-5p transfer to tumor cells, exacerbating EMT via EGF receptor (EGFR)/ERK pathway activation to drive metastasis [74]. Strategic targeting of CAF functionality represents a promising approach to overcome ADT resistance in prostate cancer.

Docetaxel and cabazitaxel remain the standard chemotherapeutic agents for metastatic PCa. CAF-derived exosomal miR-432-5p inhibits ferroptosis via targeting (ChaC glutathione-specific γ-glutamylcyclotransferase 1CHAC1), thereby enhancing docetaxel resistance in PCa [75]. CAF-expressed ANGPTL4 (angiopoietin-like protein 4) binds IQGAP1 (IQ motif-containing GTPase-activating protein 1) on PCa cells, activating the Raf-MEK-ERK-PGC1α signaling cascade. This axis stimulates mitochondrial biogenesis and oxidative phosphorylation (OXPHOS), thereby driving the chemoresistant progression of tumors [76]. Following chemotherapy, cancer-associated myofibroblasts increase the secretion of CXCL13. CXCL13 recruits B lymphocytes that release cytokines, activating nuclear factor kappa-B kinase subunit α (IKKα) in CRPC cells, promoting survival and proliferation [77]. However, whether CAFs similarly upregulate CXCL13 remains uncharacterized. Collectively, CAFs orchestrate multimodal chemoresistance mechanisms in PCa. Immunologically “cold” tumor microenvironments in PCa, characterized by limited T cell infiltration, underlie immunotherapy resistance [78]. Therefore, CAF-mediated immune evasion mechanisms through tumor-stromal crosstalk remain underexplored in PCa. In other malignancies (e.g., metastatic urothelial carcinoma, breast cancer), TGF-β signaling correlates with diminished immunotherapy response [79]. In the future, mechanistic investigations are required to delineate CAF-driven immunotherapy resistance pathways in PCa. Localized PCa management combines radical prostatectomy with radiotherapy [80]. Co-culture models demonstrate CAF radioresistance to 137Cs γ-irradiation via enhanced DNA damage repair capacity [81]. Whether CAFs confer radioresistance to adjacent tumor cells requires further investigation.

#### 3.1.5 CAF-Induced Accelerated Tumor Metastasis

Metastasis is indeed a major cause of mortality in prostate cancer, and growing evidence implicates CAFs as active facilitators of the metastatic cascade.

CAFs secrete a wide array of growth factors, cytokines, chemokines, and ECM remodeling enzymes that enhance motility, invasion, and EMT of tumor cells. For example, CAFs can produce TGF-β, CXCL12 (Stromal cell-derived factor-1, SDF-1), and interleukins, which act on cancer cells to trigger EMT programs, increase migration, and create permissive microenvironments [82,83].

Recent work also shows that autophagy in CAFs (mediated by ATG5) contributes to CAF pro-tumor functions: suppression of CAF autophagy by ATG5 knockdown reduced prostate cancer cell invasion and *in vivo* metastasis in mouse xenograft models [84].

CAFs engage in metabolic coupling with tumor cells: they may provide nutrients or metabolic byproducts (e.g., lactate, amino acids) or transfer mitochondria to cancer cells, thereby fueling OXPHOS or anabolic metabolism in metastatic clones. In PCa, CAF-cancer metabolic crosstalk promotes mitochondrial transfer and “OXPHOS addiction” in cancer cells, facilitating more aggressive phenotypes [82,85].

A particularly compelling recent study shows that after androgen deprivation, CAFs secrete exosomes enriched in miR-196b-5p, which can be transferred to PCa cells and repress the target HOXC8, thereby activating NF-κB signaling and upregulating EMT markers, thus promoting migration and metastasis [86].

Taken together, CAFs in PCa can be seen as “metastasis enablers”—not merely passive bystanders, but active participants that prime cancer cells, sculpt ECM routes, deliver metabolic support, and shape the distant microenvironment to favor metastatic outgrowth.

#### 3.1.6 CAF-Mediated Remodeling of the Extracellular Matrix

CAFs remodel ECM architecture to modulate PCa cell migration, invasion, and proliferation [87,88]. CAF-derived ECM proteins (collagen, fibronectin, tenascin C, matrix metalloproteinases/matrix metalloproteinases [MMPs]) reprogram PCa cell behavior through biomechanical and biochemical signaling. As the predominant ECM constituent, CAF-driven collagen deposition induces pathological matrix stiffening and fibrosis [89,90]. Matrix stiffening activates mechanotransduction pathways (FAK-Rac-ERK, YAP/TAZ) that drive PCa progression and invasion [90,91]. Elevated interstitial pressure from matrix remodeling reduces vascular perfusion, creating biophysical barriers that impair drug delivery and promote therapy resistance [92]. A study reveals 57.6% greater tissue stiffness in PCa vs. benign prostate tissue [93]. Fibronectin is another major ECM protein. CAF-assembled fibronectin matrices can guide directional PCa cell migration [94]. This migratory behavior is mediated by α5β1 integrin-dependent mechanotransduction, resulting in increased contractility and traction. Moreover, PDGFRα synergizes with α5β1 integrin to amplify cellular contractility. Elevated tenascin C (TNC) expression correlates with adverse clinical outcomes in PCa [95]. The matricellular glycoprotein TNC modulates PCa cell migration/proliferation via cytokine networks and oncogenic signaling [96]. However, the spatiotemporal regulation of TNC in PCa progression requires further elucidation of its mechanistic aspects. MMPs, zinc-dependent endopeptidases, facilitate tumor invasion by degrading basement membrane components [97]. MMP9 silencing reduces VEGF/ICAM-1 secretion, suppressing tumor angiogenesis [98]. MT1-MMP (Membrane type 1-MMP) facilitates osteolytic bone metastasis via Src activation triggered by NF-κB ligand from osteoblasts and PCa cells [99]. This mechanism underlies PCa’s propensity for skeletal metastasis.

### 3.2 Immunomodulatory Roles of CAFs in PCa

**Figure 2:**
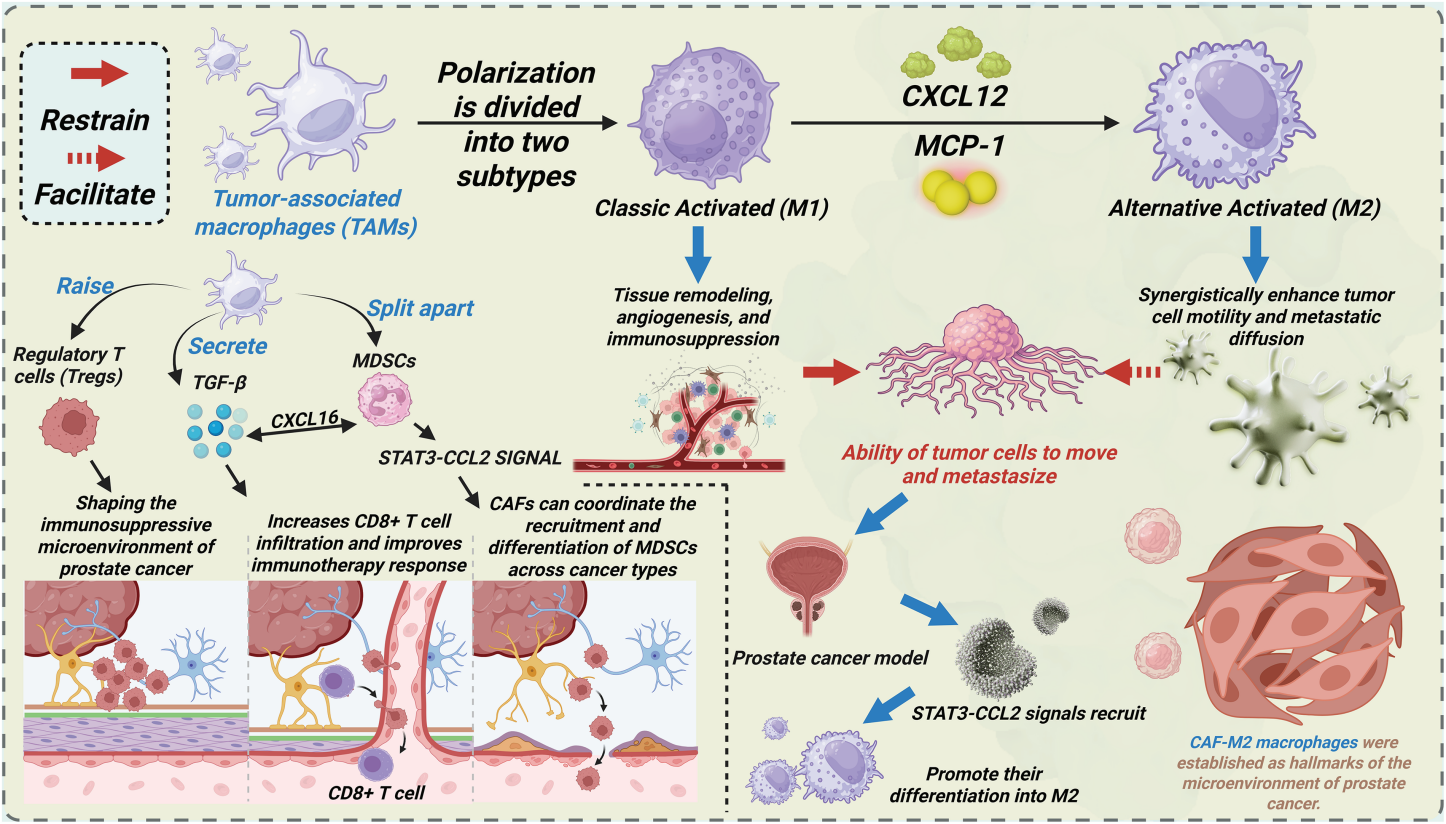
CAFs promote PCa progression via interacting with other cell types and signaling pathways within the tumor microenvironment. This figure illustrates the polarization of TAMs into two subtypes: Classic Activated (M1) and Alternative Activated (M2), with their distinct roles in PCa. The M1 subtype is associated with tissue remodeling, angiogenesis, and immunosuppression, while the M2 subtype enhances tumor cell motility and metastatic diffusion. Key signaling pathways, such as CXCL12, MCP-1, and STAT3-CCL2, mediate these processes. The figure also highlights the interaction of CAFs with TAMs and their contribution to the immunosuppressive microenvironment of prostate cancer. TAMs: Tumor-associated macrophages; CXCL16: C-X-C Motif Chemokine Ligand 16; MDSCs: Myeloid-derived suppressor cells; STAT3: Signal transducer and activator of transcription 3; CCL2: C-C Motif Chemokine Ligand 2; M1: Classic Activated macrophages; M2: Alternative Activated macrophages; CXCL12: C-X-C Motif Chemokine Ligand 12; MCP-1: Monocyte Chemoattractant Protein-1; Tregs: Regulatory T cells; CD: Cluster of differentiation

TAMs play a significant role in tumor progression (Fig. 2). Macrophages polarize into two distinct subtypes: classically activated (M1) and alternatively activated (M2). M1 macrophages secrete pro-inflammatory cytokines that enhance antitumor immunity and inhibit tumor growth [100]. Conversely, M2 macrophages drive tumor progression through tissue remodeling, angiogenesis, and immunosuppression [101–103]. In PCa microenvironments, chemokines CXCL12 and MCP-1 recruit TAMs and polarize them toward an M2 phenotype [104,105]. M2 macrophages induce EMT in CAFs, synergistically enhancing tumor cell motility and metastatic dissemination through bidirectional crosstalk. Spatial transcriptomics reveals co-localization patterns between CAFs and M2 macrophages [106]. Ligand-receptor analysis reveals that enhanced interaction occurs in co-localization zones between CAFs and M2 macrophages compared to the dominant regions of either CAFs or M2 macrophages. These findings indicate CAF-M2 macrophage interactions as a hallmark feature of PCa microenvironments.

T cells, particularly regulatory T cells (Tregs), critically shape the immunosuppressive TME in PCa [78,107,108]. CAF-driven stromal fibrosis remodels tumor vasculature through mechanical and biochemical cues. Hypoxia and chronic inflammation orchestrate Treg recruitment into the PCa TME [106]. CAF-derived TGF-β induces Treg differentiation by upregulating FOXP3/IL-2 in naïve CD4^+^ T cells [106]. Additionally, lactate from CAFs activates NF-κB, promoting FOXP3 expression during the polarization of naïve T cells toward Tregs. Immunofluorescence staining of mouse tissues and immunohistochemistry of human prostate samples demonstrate anatomical co-localization of CAFs and Tregs in PCa, and similar findings are identified in spatial transcription [106]. These data establish CAF-Treg crosstalk as a hallmark of PCa immunopathogenesis. Tregs mediate immunosuppression by inhibiting cytotoxic T cells, tumor-infiltrating lymphocytes (TILs), natural killer (NK) cells, dendritic cells (DCs), and neutrophils, thereby facilitating PCa progression [109]. In breast cancer models, CAF depletion enhances CD8^+^ T cell infiltration and improves the response to immunotherapy [110]. Whether this CAF-CD8^+^ T cell regulatory axis operates similarly in PCa requires mechanistic validation.

MDSCs, originating from hematopoietic stem cells, comprise heterogeneous populations of immunosuppressive immature myeloid cells with abnormal activation states [111]. MDSCs are morphophenotypically categorized into polymorphonuclear (PMN-MDSCs) and monocytic (M-MDSCs) subtypes [112]. MDSCs demonstrate Treg expansion, NK cell suppression, and significant prognostic value in patient survival [113–115]. While MDSC-mediated immunosuppression via T/NK cell regulation is well-characterized in PCa, the crosstalk between CAF and MDSC remains understudied. In hepatic carcinoma models, FAP^+^ CAFs recruit MDSCs via STAT3-CCL2 signaling, driving tumor progression [115]. Breast cancer CAFs secrete CXCL16 to recruit monocytes and drive M-MDSC differentiation [115]. Oxidative stress further enhances monocyte-to-MDSC differentiation [116]. Emerging evidence suggests that CAFs may orchestrate the recruitment and differentiation of MDSCs across malignancies, warranting a mechanistic investigation in PCa.

### 3.3 Angiogenic Crosstalk between CAFs and Endothelial Cells

Endothelial cells are critical mediators of angiogenesis within the PCa microenvironment [117]. CAF-derived IL-6 stimulates endothelial cell migration, neovascularization, and PCa cell proliferation [118]. IL-6 drives angiogenesis via NF-κB p50 activation and CXCL12 signaling [119,120]. Paradoxically, TNFα inhibits endothelial growth and angiogenesis by disrupting VEGF/HGF-mediated signaling [121,122]. CAF-secreted VEGF directly modulates endothelial cell function to promote tumor angiogenesis [123,124]. Interestingly, VEGF overexpression induces vascular hyperpermeability and abnormal vasculature, thereby creating physical barriers that impede immune cell infiltration [125,126]. Besides, IL-6-stimulated PCa cells exhibit autocrine VEGF secretion, amplifying angiogenic signaling [127].

### 3.4 Metabolic Interactions between CAFs and Adipocytes

Adipocytes promote stem-like properties and mesenchymal traits in PCa cells, enhancing their tumorigenicity and conferring resistance to docetaxel and cabazitaxel [128]. Within the TME, CAFs drive adipocyte transformation into tumor-promoting adipocytes through paracrine signaling [129]. Enhanced adipose lipolysis releases free fatty acids that stimulate CAF-derived IL-8 production, while triggering tumor cell secretion of MIC-1 (Macrophage Inhibitory Cytokine-1) to amplify IL-6/IL-8 signaling through autocrine loops [130]. IL-6 drives PCa proliferation via JAK-STAT, ERK1/2-MAPK, and PI3K-AKT signaling cascades [131]. IL-6 further potentiates EMT and bone metastatic progression in PCa. IL-6 recruits MDSCs and polarizes macrophages toward an M2-dominant phenotype [132]. IL-8 mediates angiogenesis and metastatic dissemination via CXCR1/CXCR2 signaling in PCa [133]. Adipocyte-secreted adipokines (e.g., adiponectin) confer radioresistance to CAFs through cytoprotective mechanisms [133]. These evidences show that extracapsular PCa invasion activates CAF-adipocyte crosstalk in periprostatic fat, accelerating disease progression through metabolic symbiosis.

## 4 Therapeutic Targeting of CAF-Mediated Interactions in Prostate Cancer

**Figure 3:**
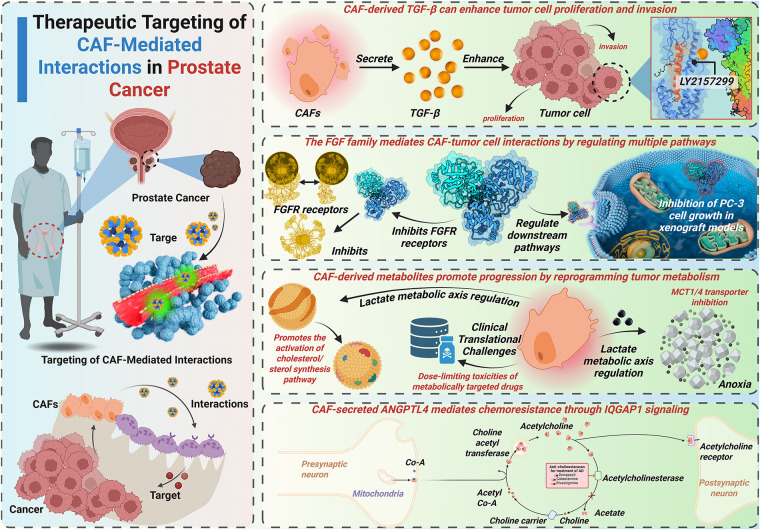
CAF-mediated TGF-β/FGF signaling, metabolic reprogramming, and therapeutic resistance in prostate cancer. This figure outlines the therapeutic strategies targeting CAFs and their interactions with PCa cells. It highlights how CAF-derived TGF-enhances tumor cell proliferation and invasion, the role of the FGF family in regulating multiple pathways, and the impact of CAF-derived metabolites on tumor progression via metabolic reprogramming. Additionally, the figure illustrates the clinical challenges of targeting CAFs in cancer therapy, including drug toxicity and resistance mechanisms mediated by CAF-secreted ANGPTL4. FGFR: Fibroblast growth factor receptor; ANGPTL4: Angiopoietin-like 4; Acetyl-CoA: Acetyl coenzyme A; LY2157299: A specific inhibitor for TGF-β receptor signaling; IQGAP1: IQ motif-containing GTPase-activating protein 1

Accumulating evidence demonstrates the critical role of the TME in PCa progression. Within the TME, CAFs have emerged as pivotal regulators through extensive crosstalk with stromal and immune components. CAFs orchestrate synergistic interactions with tumor cells, immune cells, endothelial cells, and adipocytes, perpetuating cycles of immune suppression and treatment resistance. Despite advances in therapeutic strategies, persistent treatment failures position CAFs as promising therapeutic targets [134]. Emerging preclinical models demonstrate that targeting CAF-mediated communication networks represents a novel therapeutic paradigm (Fig. 3).

### 4.1 Strategies to Inhibit CAF-Tumor Cell Signaling and Metabolism

#### 4.1.1 Targeting Growth Factor Signaling

CAF-derived TGF-β enhances tumor cell proliferation and invasiveness. Consequently, TGF-β represents a critical therapeutic target. The TGF-βR inhibitor LY2109761 suppresses CAF-mediated PCa cell proliferation/migration *in vitro* [44]. Galunisertib (LY2157299), a TGF-βRI inhibitor, sensitizes PCa cells to enzalutamide [135]. Nitazoxanide inhibits TGF-β-mediated bone metastasis *in vivo*, which is primarily indicated for parasitic/viral infections [136,137]. Mechanically, TGF-β induces KLF5 acetylation in PCa bone metastases, driving docetaxel resistance [138]. The FDA-approved drug plerixafor, targeting KLF5/CXCR4 signaling, mitigates TGF-β-induced chemoresistance. Current TGF-β-targeted strategies primarily focus on TGF-β receptors or downstream effectors. Direct TGF-β inhibition remains underexplored and requires further investigation. Targeting TGF-β-mediated crosstalk between CAFs and tumor cells may yield novel therapeutic approaches.

The FGF family critically mediates CAF-tumor cell crosstalk by modulating multiple signaling pathways. Analogous to TGF-β targeting, therapeutic strategies against FGF signaling involve FGF receptor (FRFR) inhibition and downstream pathway modulation. Emerging approaches additionally target FGF ligands directly. FGFR inhibitors exhibit broad-spectrum efficacy against CRPC models *in vitro* [139]. The FGFR inhibitor SSR128129E (SSR) binds extracellular domains, demonstrating multi-receptor inhibition with enhanced therapeutic index [140]. Notably, SSR exhibits oral bioavailability, establishing allosteric FGFR targeting as a novel paradigm for drug development. FGF-binding protein (FGF-BP) acts as a molecular chaperone to amplify FGF-2 activity in PCa microenvironments [141]. Ribozyme-mediated FGF-BP knockdown ablates PC-3 cell growth *in vivo* using xenograft models [142]. FGF5 coordinates with Hedgehog signaling to drive mitogenic programs in PCa [56]. Systematic Evolution of Ligands by EXponential (SELEX)-based screening identified an FGF5-specific RNA aptamer with strong affinity [143]. This FGF5-targeted aptamer shows superior specificity and reduced off-target effects compared to FGFR inhibitors, highlighting ligand-specific targeting as a precision therapeutic approach.

#### 4.1.2 Disrupting Metabolic Crosstalk

CAF-derived metabolites drive PCa progression through metabolic reprogramming. These metabolic vulnerabilities present novel therapeutic targets. CAF-secreted lactate critically depends on MCTs for intercellular shuttling. MCT1/4 silencing inhibits PCa progression under hypoxic and androgen-deprived conditions. Targeting lactate flux via MCT4 inhibition or lactate dehydrogenase A (LDHA) blockade radiosensitizes tumors and enhances the efficacy of chemotherapeutic agents [144]. Phase I trials demonstrate oral bioavailability of the MCT1 inhibitor AZD3965 (10 mg twice daily) in advanced solid tumors and lymphomas [145]. Clinical validation of AZD3965 in PCa-specific cohorts remains pending. CAF-derived glutamine elevation correlates with ADT resistance in PCa [67]. Despite the dose-limiting toxicity of the glutamine antagonist 6-diazo-5-oxo-l-norleucine (DON), its prodrug, DRP-104, can transform into DON in tumor cells, thereby decreasing its toxicity [146]. DRP-104 dual-targets glutamine carbon/nitrogen metabolism, inducing apoptosis and suppressing CRPC growth [146]. Metformin, a drug used in patients with type II diabetes, induces metabolic vulnerability in PCa by inhibiting glucose oxidation and forcing reliance on reductive glutamine metabolism [147]. This metabolic synthetic lethality suggests therapeutic synergy between glutamine antagonists and metformin in PCa. The glutaminase inhibitor CB-839 oral formulation targets glutamine biosynthesis, a key metabolic node in PCa [148]. Phase I trials demonstrate CB-839’s safety profile and preliminary efficacy in advanced solid malignancies [148]. PCa-specific clinical evaluations of CB-839 remain limited.

#### 4.1.3 Epigenetic Modulation of CAFs

Given the conceptual and indirect evidence, targeting CAFs through epigenetic modulation in prostate cancer may be feasible. One approach would be to use DNA methyltransferase inhibitors (DNMTi) or histone deacetylase inhibitors (HDACi) (or other “epi-drugs”) to reverse aberrant methylation or histone deacetylation in CAFs, thereby diminishing expression of tumor-promoting genes (e.g., cytokines, ECM remodeling enzymes). Because many of these compounds are already in use or clinical trials in PCa (targeting tumor cells), repurposing or dual targeting (cancer + stroma) might be possible [149,150].

#### 4.1.4 Inhibiting Key Downstream Oncogenic Pathways

CAF-activated PI3K/AKT signaling in PCa cells drives resistance to ADT. The PI3K inhibitor LY294002 reverses ADT resistance in preclinical models [72]. The AR inhibitor enzalutamide (MDV3100) and PI3K inhibitor buparlisib (BKM120) exhibit synergistic effects in restoring androgen sensitivity [151]. However, Phase II trials demonstrated no significant clinical benefit of buparlisib in reversing metastatic CRPC (mCRPC) progression [152]. The PI3K inhibitor ipatasertib, combined with abiraterone, showed an improved outcome in a multicenter, randomized, double-blind, phase III trial [153]. Phase II trials revealed that the capivasertib-enzalutamide combination was well-tolerated but failed to improve outcomes in abiraterone-pretreated mCRPC [154]. Capivasertib added to docetaxel/prednisolone did not prolong composite progression-free survival in mCRPC patients in a phase II trial [155]. Further analysis suggested a potential benefit of capivasertib-docetaxel in abiraterone/enzalutamide-pretreated mCRPC cohorts [156]. Generally, PI3K/AKT pathway-targeted therapies remain under active clinical investigation in PCa [157]. CAFs induce AKR1C3 overexpression in PCa cells, enhancing intratumoral cholesterol/steroid biosynthesis [73]. AKR1C3 activation correlates with enzalutamide resistance, while its inhibitor, indomethacin, shows partial reversal efficacy in CRPC models [158]. However, Phase I/II trials demonstrated limited clinical efficacy of indomethacin in CRPC patients [159]. CAF-derived exosomal miR-146a-5p inhibits EGFR, thereby constraining PCa progression. Conversely, ADT-induced loss of miR-146a-5p promotes metastasis [74]. While direct miR-146a-5p targeting remains underdeveloped, EGFR inhibition has emerged as a promising therapeutic strategy. The E3 ubiquitin ligase FBXW2 mediates EGFR ubiquitination and degradation, suppressing EGF-driven PCa growth [160]. The EGFR tyrosine kinase inhibitor arglabin demonstrates anti-proliferative effects in PCa preclinical models [161]. The EGFR monoclonal antibody cetuximab combined with docetaxel showed manageable toxicity and competitive efficacy in mCRPC phase II trials [162]. EGFR remains a biologically validated target, warranting the development of optimized therapeutic approaches for treatment-resistant PCa.

#### 4.1.5 Targeting ECM Remodeling and Physical Barriers

CAF-secreted ANGPTL4 drives PCa chemoresistance via IQGAP1-mediated signaling [76]. The IQGAP1 inhibitor QGGP reverses ANGPTL4-mediated chemoresistance in preclinical models. These data suggest that ANGPTL4 may be a potential therapeutic target in chemoresistant PCa. ANGPTL4 silencing attenuates PCa cell proliferation *in vitro* [163]. However, the translational development of ANGPTL4-targeted therapies remains in its early stages. CAF-mediated ECM remodeling drives prostate cancer progression via FAK/Rac/ERK and YAP/TAZ signaling pathways, representing promising therapeutic targets. Pharmacological inhibition of FAK suppresses YAP activation and inhibits the progression of CRPC [164]. Clinical-stage FAK inhibitors (GSK2256098, PF-00562271, VS-6063, BI 853520) demonstrate emerging therapeutic potential for CRPC treatment [165]. The YAP/TAZ inhibitor verteporfin reverses castration resistance and inhibits bone metastasis in CRPC models [166]. Moreover, CAF-induced ECM stiffening creates biophysical barriers that restrict drug penetration and distribution. Therefore, developing novel and efficient drug delivery systems is also crucial for improving the outcome of CRPC therapy. Anisamide-targeted nanoparticles (~18 nm) exhibit 7-fold greater CAF accumulation compared to non-target cells at 16 h post-injection [167]. Another FAPα-targeted cell-penetrating peptide-nanoparticle enables efficient siRNA delivery to CAFs, reducing CXCL12-mediated metastasis [168]. Besides, the Extradomain-B fibronectin-specific peptide enables molecular imaging of PCa metastases [169]. However, therapeutic targeting of fibronectin remains underdeveloped. MMPs drive PCa metastasis through the degradation of the ECM [170]. Curcumin inhibits MMP2/9 activity, significantly reducing metastatic nodules *in vivo* [170]. Similar to curcumin, atorvastatin downregulates MMP expression, suppressing PCa cell migration/invasion *in vitro* [171]. In Phase I/II trials, marimastat, an MMP inhibitor, was found to effectively delay biochemical recurrence in PCa patients [172]. Batimastat, another kind of MMP inhibitor, demonstrated similar anti-metastatic efficacy [173]. First-generation MMP inhibitors failed phase III trials due to multiple causes [174]. Next-generation MMP inhibitors with enhanced selectivity are being developed to overcome the limitations of previous generations of inhibitors.

In general, therapeutic strategies targeting CAF-tumor cell crosstalk focus on signal transduction and metabolic interference. The TGF-β and FGF families, as well as lactate and glutamine, are the most widely discussed targets. Additionally, recent research focuses on overcoming therapeutic resistance through ECM remodeling strategies and inhibiting signaling pathways.

### 4.2 Modulation of CAF-Immune Cell Crosstalk to Restore Anti-Tumor Immunity

The dynamic interplay between CAFs and immune cells within the TME critically drives PCa progression. CAF-secreted cytokines and chemokines orchestrate immune cell recruitment, polarization, and functional reprogramming to establish an immunosuppressive environment. Targeting CAF-immune cell interactions represents a strategic approach to overcoming immune evasion and treatment resistance. Among all immune cells involved in PCa, TAMs, Tregs, and MDSCs may emerge as key mediators of PCa pathobiology.

Therapeutic strategies targeting TAMs include reducing the recruitment of TAMs, disrupting the interaction between TAMs and CAFs, and preventing the polarization of TAMs to the M2 phenotype mediated by CAFs. CCL2, also called MCP-1, is a crucial cytokine in recruiting TAMs, and its inhibitor, Carlumab, is supposed to be beneficial to PCa patients. However, phase II clinical trials revealed no significant tumor regression with carlumab in mCRPC [175]. CAF-derived CXCL12/CXCR4 signaling drives TAM recruitment and M2 polarization through STAT3 activation. The CXCR4 antagonist CTCE-9908 inhibits metastatic dissemination in preclinical PCa models [176,177]. Other therapeutic strategies, including CXCR4 antagonists, CXCR4 antibodies, and medicines targeting CXCL12, also demonstrate pan-cancer efficacy [178]. Emerging TAM-reprogramming agents include phosphatidylserine-targeting antibody 2aG4, simvastatin, and zoledronic acid [111]. However, a systematic investigation is required to determine whether CAFs play a role in the repolarization of TAM after being treated with these TAM-reprogramming agents.

Current T cell-directed therapies predominantly target immune checkpoints like Cytotoxic T lymphocyte-associated antigen-4, CTLA-4 and Programmed death receptor 1/Programmed death ligand 1 (PD-1/PD-L1). Checkpoint inhibitors reinvigorate exhausted T cells by blocking CTLA-4/CD80 and PD-1/PD-L1 interactions. However, CAF-mediated modulation of checkpoint inhibitor efficacy remains poorly characterized. Due to our limited understanding of how CAFs influence T cell exhaustion and Treg expansion, next-generation immunotherapies targeting the crosstalk between CAFs and T cells are still in development.

MDSC biology in PCa similarly presents therapeutic challenges and opportunities. However, previous studies have shown that VEGF and CXCL5/CXCR2 are strongly associated with the recruitment of MDSCs in other kinds of tumors [179,180]. Cabozantinib (VEGFR inhibitor) and CXCR2 antagonists can reduce MDSC infiltration [181,182]. We speculate that CAF-derived VEGF/CXCL5 may coordinate MDSC recruitment via paracrine signaling. Systematic mapping of CAF-immune cell interactomes will advance the development of precision immunotherapy in the future.

### 4.3 Targeting CAF-Endothelial Interactions to Normalize Tumor Vasculature

The PD-1/PD-L1 immune checkpoint axis is a key mediator of tumor immune evasion in prostate cancer [183]. However, limited clinical response rates highlight the need for improved immunotherapy strategies. CAF-derived VEGF drives pathological angiogenesis through endothelial activation and vascular abnormalization. Preclinical studies demonstrate that anti-VEGF monotherapy inhibits endothelial tube formation *in vitro*, prompting the development of the TGF-β/VEGF bispecific antibody Y332D [184]. Y332D synergizes with PD-1 blockade to enhance local lymphocyte density and functionality, restoring anti-tumor immunity. Other clinical-stage VEGF inhibitors demonstrate variable efficacy in PCa. Bevacizumab is a humanized monoclonal antibody targeting VEGF. A case report observed PSA reduction in a CRPC patient who received bevacizumab [185]. AZD2171, an inhibitor of VEGFR1/2, has been proven to be effective in hormone-refractory prostate cancer (HRPC) in a Phase I dose escalation and pharmacokinetic study [186]. Sunitinib, a VEGFR-2 inhibitor, is also competitive for CRPC due to the analysis of a phase II study [187]. However, not all anti-VEGF drugs have good clinical trial results. Pazopanib is identified as controversial in castrate-sensitive PCa during a randomized, phase II study due to its toxicities [188]. However, challenges also exist in these clinical trials. Generally, vascular normalization through modulation of the CAF-endothelial axis may overcome immunotherapy resistance in the future.

### 4.4 Interrupting CAF-Adipocyte Metabolic Symbiosis

IL-6 and IL-8 serve as key mediators orchestrating CAF-adipocyte crosstalk through paracrine signaling. A phase I trial demonstrated that siltuximab (a monoclonal antibody against IL-6) combined with docetaxel achieved a preliminary efficacy in CRPC patients [189]. Another phase II trial showed no improved outcome with siltuximab plus mitoxantrone/prednisone in CRPC [190]. HuMax-IL8, also called BMS-986253, is a monoclonal antibody against IL-8. Proved by a phase I trial, it is safe and well-tolerated in patients with metastatic or unresectable locally advanced solid tumors, including PCa [191]. More clinical trials are ongoing to investigate the combination of IL-8 blockade and other immunotherapies.

## 5 Conclusions and Future Perspectives

PCa ranks among the most prevalent and life-threatening cancers affecting male populations worldwide. Once afflicted with this androgen-driven malignancy, patients will accept combined treatment strategies. However, if PCa progresses to the metastatic stage, a satisfactory therapeutic effect cannot be achieved. CRPC remains a fatal stage with limited options. The TME is a critical determinant of PCa progression and treatment resistance. Recently, CAFs have emerged as central orchestrators of PCa tumorigenesis, immune evasion, and therapeutic resistance within the TME.

### 5.1 Recapitulation of CAF Complexity and Therapeutic Challenges

**Figure 4:**
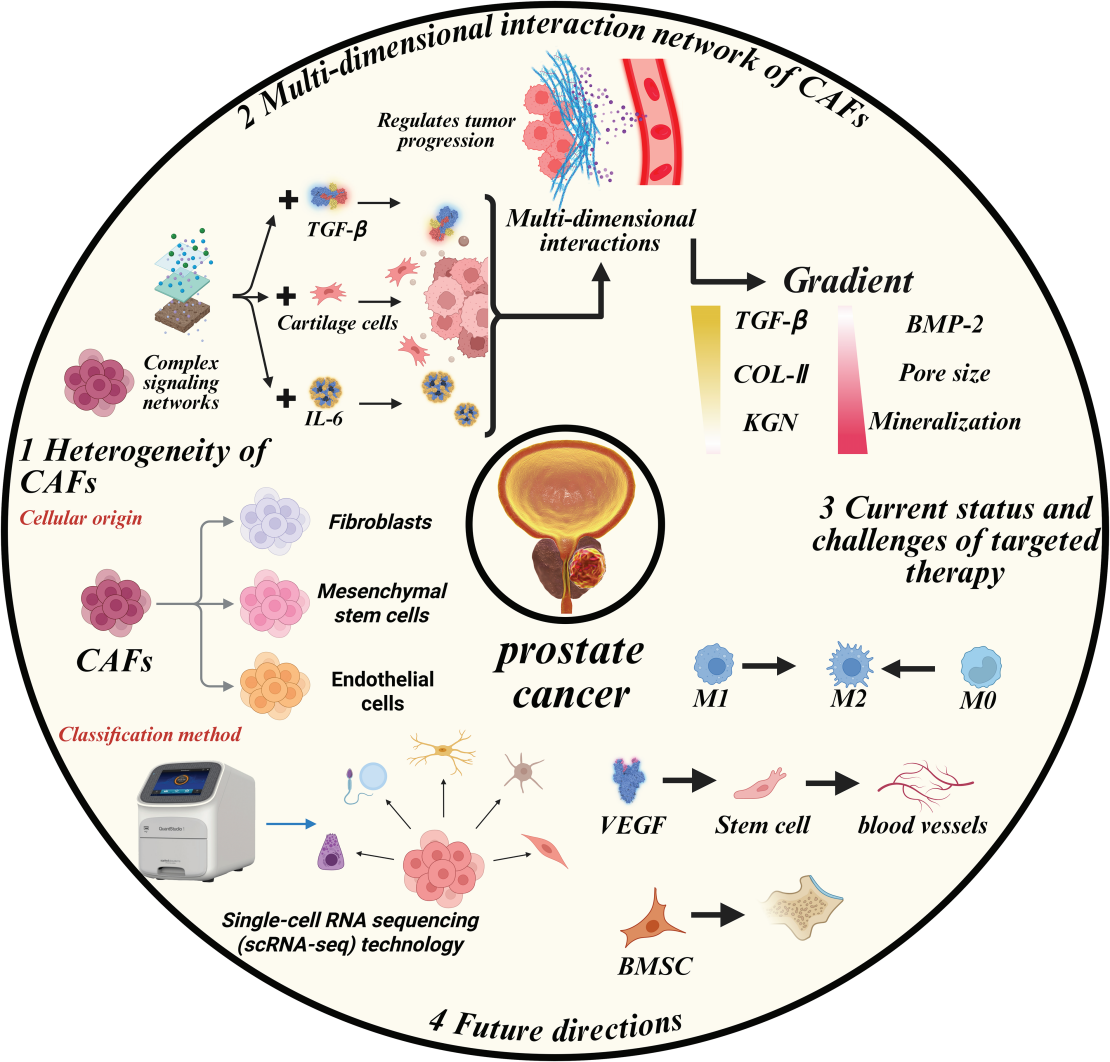
CAFs orchestrate PCa progression via multidimensional crosstalk. This figure illustrates the heterogeneity of CAFs based on their cellular origin, including fibroblasts, mesenchymal stem cells, and endothelial cells. It also highlights the complex signaling networks and interactions involving TGF-β, IL-6, and cartilage cells that regulate tumor progression. Additionally, the figure shows the gradient of key molecules, including TGF-β, COL-II, and BMP-2, that influence cancer progression. The current status and challenges of targeted therapy, such as M1, M2, and M0 macrophage polarization, are also illustrated, along with future directions of cancer research and therapy, including single-cell RNA sequencing (scRNA-seq) technology and stem cell research. IL-6: Interleukin 6; VEGF: Vascular endothelial growth factor; M0: Resting macrophages; COL-II: Collagen type II; BMP-2: Bone morphogenetic protein 2; KGN: Kartogenin; BMSC: Bone marrow-derived stem cells

CAFs comprise heterogeneous fibroblast populations with diverse cellular origins and functional phenotypes. Accumulating evidence can reveal CAF origins from fibroblasts, MSCs, endothelial cells, EMT, and adipose tissues. Most researchers use a function or marker as a means of identification to classify CAFs, and scRNA-seq enables further classification of CAF subtypes, thereby facilitating scientific research. To better understand the role of CAFs in the progression of PCa, we synthesize the multidimensional crosstalk between CAFs with tumor cells, immune cells, endothelial cells, and adipocytes in TME. We further evaluate therapeutic strategies targeting CAF-mediated interactions to overcome treatment resistance and improve clinical outcomes (Fig. 4).

CAF-tumor cell interaction is the most extensively studied field, with a focus on the proliferation ability and treatment resistance of tumor cells. Additionally, CAF-mediated ECM remodeling establishes biomechanical niches that promote metastatic progression. CAFs orchestrate immunosuppression through coordinated interactions with TAMs, Tregs, and MDSCs. Angiogenic CAF-endothelial crosstalk and adipocyte metabolic reprogramming represent emerging research frontiers. Based on previous studies, researchers are seeking novel therapeutic approaches that target key points of the dynamic interaction between CAFs and other cells in the TME of PCa, aiming to break the vicious cycle. Several clinical trials have been done to confirm their safety and efficiency. Some new attempts are successful, while others fail to achieve a satisfactory outcome. Despite being classified as influential in the interaction between CAFs and a specific cell type, core signaling nodes (e.g., TGF-β, IL-6) exhibit pleiotropic effects across multiple CAF interaction networks. TGF-β primarily mediates CAF-tumor interactions but secondarily modulates CAF-endothelial crosstalk. IL-6 plays critical roles in both CAF-endothelial crosstalk and CAF-adipocyte crosstalk. The CAF interactome in PCa TME functions as a multidimensional signaling network, rather than a linear pathway. Therefore, it remains a considerable distance from fully understanding the pathogenic mechanism of CAFs in PCa.

Unfortunately, there is still no clinical treatment targeting CAFs. Several obstacles may hinder the clinical application of CAF-targeted therapies. CAFs are highly heterogeneous, displaying overlapping markers with normal fibroblasts, myofibroblasts, and macrophages. This complicates the identification of CAF-specific targets without affecting normal stromal functions. Inconsistent subtype definitions across studies further impede therapeutic standardization. Also, CAFs orchestrate a multidimensional network involving tumor cells, immune cells, endothelial cells, and adipocytes. Targeting a single pathway (e.g., TGF-β, IL-6, or FGF) may be insufficient due to redundant signaling and compensatory feedback loops. Hence, combinatorial targeting strategies or systems-level modeling may be necessary. Moreover, many CAF-related pathways (e.g., TGF-β, FGF, PI3K/AKT) are essential for normal tissue homeostasis and repair. Systemic inhibition of these pathways risks adverse effects, fibrosis, or immune dysregulation, raising safety concerns for clinical translation.

### 5.2 Limitations of Current Research and Methodological Advancements

Despite providing a comprehensive overview of the role of CAFs in PCa progression and therapy resistance, several limitations should be acknowledged. Most data summarized in this review derive from *in vitro* co-culture systems or murine xenograft models. Although these models elucidate mechanistic interactions between CAFs and tumor or immune cells, they may not fully recapitulate the spatial complexity and dynamic heterogeneity of the human prostate TME. Also, most CAF analyses rely on single time-point tumor biopsies, missing the temporal evolution of CAF phenotypes during androgen-deprivation therapy or chemotherapy. Longitudinal sampling with spatial transcriptomics could better define dynamic CAF reprogramming. In response to these unsatisfactory aspects, organoid and ST technology may be a supplement. Orgnoid models preserve genomic and stromal integrity better than 2D cultures, improving predictive value for CAF–tumor crosstalk. Incorporating ST enables mapping of CAF–immune–tumor cell interactions within their native context. As more and more of these techniques are applied to study CAF in PCa, the research results may be more convincing.

In recent years, several high-impact studies have provided new insights into the clinical significance and therapeutic potential of CAFs in PCa, underscoring their pivotal role in disease progression and therapy resistance. A landmark study published in Cancer Cell (2023) demonstrated that ADT induces a profound reprogramming of stromal fibroblasts, leading to the emergence of SPP1^+^ myofibroblastic CAFs that actively promote CRPC development [192]. This finding provided direct evidence that clinical anti-androgen therapy can reshape the TME and generate pro-tumorigenic CAF phenotypes, highlighting the necessity of targeting the stromal compartment alongside epithelial cancer cells. Similarly, a multicenter translational study by Talia et al. (2024) identified CAF-related gene signatures that predict clinical outcomes in prostate tumor patients [193]. Patients exhibiting high CAF-signature expression profiles had significantly shorter biochemical recurrence-free and metastasis-free survival, establishing CAF-derived transcriptomic markers as potential prognostic tools for precision stratification. Complementing these findings, a single-cell transcriptomic analysis by Shan et al. (2025) revealed distinct fibroblast subpopulations within PCa tissues and identified COMT as a key fibroblast-associated gene whose elevated expression correlates with aggressive tumor behavior and poor prognosis [194]. These recent clinical and mechanistic advances emphasize that disrupting CAF-mediated signaling networks may represent a promising adjunctive strategy to overcome resistance and improve outcomes in advanced PCa.

Overall, targeting CAF interaction networks offers a novel strategy for overcoming treatment resistance in advanced prostate cancer. Future research should combine advanced TME targeting strategies with an in-depth investigation of the complex pathogenic mechanisms in PCa.

## Data Availability

Not applicable.
